# Studying and modulating schizophrenia-associated dysfunctions of oligodendrocytes with patient-specific cell systems

**DOI:** 10.1038/s41537-018-0066-4

**Published:** 2018-11-19

**Authors:** Florian J. Raabe, Sabrina Galinski, Sergi Papiol, Peter G. Falkai, Andrea Schmitt, Moritz J. Rossner

**Affiliations:** 1 Molecular and Behavioural Neurobiology, Department of Psychiatry and Psychotherapy, University Hospital, LMU Munich Munich, Germany; 2 International Max Planck Research School for Translational Psychiatry (IMPRS-TP), Munich, Germany; 3 Institute of Psychiatric Phenomics and Genomics (IPPG), University Hospital, LMU Munich Munich, Germany; 4 Laboratory of Neuroscience (LIM27), Institute of Psychiatry, University of Sao Paulo, Sao Paulo, Brazil

## Abstract

Postmortem studies in patients with schizophrenia (SCZ) have revealed deficits in myelination, abnormalities in myelin gene expression and altered numbers of oligodendrocytes in the brain. However, gaining mechanistic insight into oligodendrocyte (OL) dysfunction and its contribution to SCZ has been challenging because of technical hurdles. The advent of individual patient-derived human-induced pluripotent stem cells (hiPSCs), combined with the generation of in principle any neuronal and glial cell type, including OLs and oligodendrocyte precursor cells (OPCs), holds great potential for understanding the molecular basis of the aetiopathogenesis of genetically complex psychiatric diseases such as SCZ and could pave the way towards personalized medicine. The development of neuronal and glial co-culture systems now appears to enable the in vitro study of SCZ-relevant neurobiological endophenotypes, including OL dysfunction and myelination, with unprecedented construct validity. Nonetheless, the meaningful stratification of patients before the subsequent functional analyses of patient-derived cell systems still represents an important bottleneck. Here, to improve the predictive power of ex vivo disease modelling we propose using hiPSC technology to focus on representatives of patient subgroups stratified for genomic and/or phenomic features and neurobiological cell systems. Therefore, this review will outline the evidence for the involvement of OPCs/OLs in SCZ in the context of their proposed functions, including myelination and axon support, the implications for hiPSC-based cellular disease modelling and potential strategies for patient selection.

## Introduction

Schizophrenia (SCZ) is a severe, disabling neuropsychiatric disorder with a lifetime prevalence of 0.3%–2.3%.^1–4^ The substantial disability associated with this disorder, together with its early onset and chronicity, places an enormous burden on patients. This burden was quantified in the Global Burden of Disease Study 2013, which found a remarkably high number of disability-adjusted life years (DALY) and years lived with disability (YLD) in SCZ compared with other medical conditions.^5–7^ The clinical features of SCZ have been subdivided into positive, negative and cognitive symptoms. Positive symptoms are likely to be associated with a hyper-dopaminergic state. Neuroleptics that act mainly at the dopamine receptor 2 (DRD2), which is highly expressed in the basal ganglia, are effective in reducing positive symptoms in many patients.^8^ An increasing body of evidence indicates that negative and cognitive symptoms are functionally associated with an excitation/inhibition dysbalance in the function of glutamatergic and ɣ-aminobutyric acid (GABA)ergic synapses. This dysbalance is proposed to eventually lead to a disconnection of critical cortico-cortical and cortico-subcortical projection systems, although the underlying mechanisms are not fully understood.^9^ No effective pharmacological treatment is available for negative and cognitive symptoms.^8^

So far, all attempts to target the synapse-associated glutamatergic system have failed and thus other mechanisms need to be explored to identify novel treatment options.^10^ The lack of human neurobiological test systems to study the consequences of genetic and pharmacological perturbation, for example, has been a major limitation in the field of psychiatry to date. Research has been mainly restricted to peripheral tissues, such as blood, correlative imaging studies, genetics and molecular and histological analyses of postmortem brain samples. Taken together, these approaches have revealed strong evidence for a synaptic dysfunction. In addition, current evidence of altered white matter (WM) structures and reduced myelin gene expression in SCZ suggests that dysfunctional oligodendrocyte precursor cells (OPCs) and/or oligodendrocytes (OLs) may also contribute to the dysconnectivity of brain regions seen in this disorder.^11–14^ Moreover, despite compelling advances in the understanding of genetic factors that contribute to the risk for SCZ, little is still known about the final mechanistic consequences of such risk factors in the biological systems involved in the genesis of SCZ.^15–17^

The unavailability of proper model systems to accurately assess the functional consequences for and contribution of dedicated molecular and cellular signatures to these disorders is one possible reason for the lack of biological insight. Animal models, especially inbred genetic mouse models, are well-suited to characterize the molecular and phenotypic (i.e. behavioural) impact of one or a few risk genes but cannot capture the complex genetic risk architecture of SCZ. However, recent developments in the methodologies for the induction of pluripotent stem cells and their differentiation into neuronal and glial cell types have paved the way for the generation of patient-specific cellular systems.^18–20^ These systems hold promise for comparing cellular phenotypes in patients and healthy controls, whereas keeping the individual genomic risk background of each individual. Likewise, such cellular models have some characteristics that make them especially well-suited to assessing how pharmacological treatments may restore affected pathways in patient-specific cell types.^21,22^

This review aims to (i) revisit dysfunctions of OPCs/OLs in SCZ, including and beyond myelination, and (ii) discuss strategies for patient selection towards stem-cell generation and cellular modelling as a pre-requisite to target OPC/OL deficits with pharmacological approaches.

## Oligodendrocyte dysfunction and white matter deficits in schizophrenia

### Neurodevelopment, myelination and axonal support

Puberty and adolescence are vulnerable periods of brain development and are characterized by a multitude of psychosocial challenges related to work and interpersonal relationships. Adolescence coincides with the average age of onset of affective and non-affective psychoses.^23^ The hypothesis that SCZ is not a neurodegenerative but a neurodevelopmental disease that alters brains circuits was proposed nearly 40 years ago^24,25^, but was recently rediscovered and is gaining increasing influence.^26–29^

An important process that takes place during early postnatal human brain development is myelination. The development of WM tracts occurs at a high rate in the first years of childhood and continues during puberty and even young adulthood.^30,31^ Postmortem analyses of human brain tissues have revealed that myelination follows a defined chronological and topographical order along the caudal to rostral axis.^32,33^ The fact that associative cortical areas such as the prefrontal cortex are among the latest to become myelinated, i.e. not until early adulthood, and are generally less myelinated has potential implications for SCZ.^14^ The developmental aspects of myelination that may be affected in SCZ remain largely unclear, however, because most studies have been performed in adult postmortem samples.^34^ Defects in myelination during development could be caused by a failed or delayed differentiation of the OPC lineage and/or by failed or delayed OL maturation. On the other hand, myelin deficits in SCZ could also reflect failures in the adaptive mechanism termed ‘myelin plasticity’. It has been known for a long time that electrical activity stimulates myelination and that many structural aspects of myelin-axon interactions can modulate for example conduction velocity, including myelination per se; myelin thickness; the spacing and size of the nodes of Ranvier and the clustering of ion channels.^35^ It has recently been established that several of these mechanisms are plastic and likely to contribute to the overall plasticity of the brain.^36^ In addition, segmented or partial myelination and the pattern and relationship of myelination by individual OLs of functionally distinct or potentially synchronized axons (coupled myelin bundles) have been mentioned as being important, as yet unanswered questions in the field that add additional opportunities for remodelling and plasticity.^37^ Finally, disturbances in myelination impair connectivity and coherence between brain regions by causing improper spike timing and desynchronized signal processing.^38,39^ Thus, the consequences of deficits in developmental and adaptive myelination align well with the prevailing hypothesis of schizophrenia as a ‘dysconnectivity disease’.^40^ More recently, OLs have been proven to metabolically support axons,^41,42^ an essential biological mechanism that is important for brain function and that can be uncoupled from myelination. Myelination coincides with the maturation of brain networks, which is mainly driven by the integration of interneurons into local cortical microcircuits. It has thus been hypothesized that, in addition to optimizing action potential fidelity, the segmented myelination of fast-spiking parvalbumin-positive (Parv+) interneurons may help to cope with the high energy demands of these cells.^43^ Strikingly, in the cortex of rodents most myelinated axons of interneurons belong to the Parv+ subtype.^44^ The suggestion that dysfunctions of Parv+ interneurons are associated with SCZ also led to the idea that failure of both processes, i.e. optimizing of action potential fidelity and metabolic support, may together precipitate the network dysfunctions associated with SCZ.^45^ However, this view was recently challenged because the most comprehensive analysis of the contribution of cell type-associated SCZ risk variants so far did not point to an involvement of Parv+ interneurons in the aetiology of SCZ, but rather highlighted the role of reelin-positive cortical interneurons.^46^ Also, neither OPC- nor OL-specific gene sets were enriched in SCZ risk genes identified in genome-wide association studies (GWASs).^46^ Although these observations cannot be considered as definitive, they suggest that OL dysfunctions in SCZ may rather be a secondary consequence of genetic risk factors operating primarily in glutamatergic neurons. This does not, however, exclude ‘myelin plasticity’ as a potential target for research into treatment options. On the other hand, a study where glial precursors isolated from childhood-onset SCZ patients were transplanted into mice also provided evidence for a direct and ‘primary’ contribution of OLs to SCZ^47^ (see below for details).

### Disturbed oligodendrocyte function and myelin: impact on cognition in schizophrenia

A series of studies with different techniques provided much evidence for disturbed OL function and myelin deficits in schizophrenia, which has been reviewed in detail elsewhere.^12,34^ In short, in vivo brain imaging studies revealed decreased fractional anisotropy as a sign of impaired WM tract integrity,^48,49^ a lower myelin water fraction in the WM of the frontal lobe and corpus callosum^50^ and functional dysconnectivity in relevant neuronal networks,^51^ which is supported by electrophysiological measurements.^40^ Studies in SCZ patients that combined imaging and neurocognitive testing revealed associations between impaired cognitive performance and decreased fractional anisotropy,^52,53^ disturbed connectivity^54,55^ and reduced volume of specific brain areas (hippocampus and nucleus accumbens).^56^ Furthermore, histopathological findings in postmortem brain tissue indicated a decreased number of OLs in the dorsolateral prefrontal cortex (DLPFC) and cornu ammonis (CA) 4 region of the anterior and posterior hippocampus,^57–59^ and reduced OL number was correlated with cognitive dysfunction.^60^ Moreover, a reduced density of perineuronal OLs,^61^ lower myelin basic protein (MBP) immunohistochemical staining intensities^62^ and damaged myelin sheaths and apoptosis of OLs^63,64^ have also been documented in light microscopy and electron microscopy studies. Transcriptomic studies in postmortem tissues reported reduced expression of myelin- and OL-related genes, such as MAG and MBP, in several relevant brain regions,^65,66^ and myelin-associated proteins have been shown to be decreased in proteomic studies.^67,68^

Taken together, accumulating evidence suggests that disturbances of OL function and myelination likely contribute to the dysconnectivity of brain regions and cognitive impairments seen in SCZ. Whether the deficits in OL functions and their impact on cognition is a primary causative mechanism or a secondary consequence of neuronal and synaptic alterations, or both, is still unclear, although evidence exists for both options.^46,47^

## Genetic and cellular complexity of SCZ - new interpretations from the latest GWASs and scRNAseq analyses

GWASs and exome sequencing approaches have provided outstanding and solid results regarding the role of common and rare genetic variations in psychiatric disorders, such as SCZ. In SCZ, an increasing number (so far around 150) of genetic risk *loci* have been unequivocally identified after controlling for multiple testing and for sources of confounding, such as population stratification.^16,17,69^ Likewise, these studies have provided compelling evidence of the polygenic architecture of this disorder. Polygenic risk scores (PRSs) can be calculated for everyone and summarize in a single risk score the effects of many single nucleotide polymorphisms (SNPs) under an additive model. PRSs have shown an excellent replicability across independent samples of SCZ patients, although they lack predictive value as regards disease risk.^70^

An important proportion of *loci* associated with SCZ contain at least one expression quantitative trait locus (eQTL) for a gene within 1 Mb. Active brain enhancers are enriched in these associations, highlighting the regulatory nature of such associations with SCZ risk. Further analysis revealed that several risk SNPs are associated with genes of known regulatory function in neurons and also with genes relevant for glial cells and OPCs/OLs.^16,17,71–73^ SCZ risk variants are enriched within genomic regions that are marked by histone H3-K4 methylation (H3K4me3), which points to the impact of processes related to the control of gene expression.^73^ Pathway analyses have shown the aggregation of risk variants in pathways related to neuronal signalling, postsynaptic density, FMRP-bound transcripts and mitochondrial and glial function.^74,75^ Interestingly, Duncan et al. revealed a stronger association of an expert-curated glia-OL pathway (comprising 52 genes) with SCZ than with bipolar disorder.^74^ Worth mentioning in this context is the fact that, numerically, most pathway analyses underline the best evidence of disturbed neuronal function in SCZ.

**Fig. 1:**
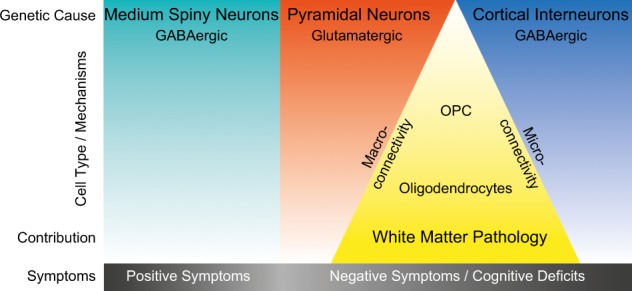
Primary and secondary cell types and mechanisms of schizophrenia (SCZ)—combining results of the latest genome-wide association studies (GWASs) with cell type-specific transcriptomics. Most recent GWASs combined with single-cell RNAseq profiles identified SCZ risk genes that may primarily operate in three neuronal cell types: GABAergic medium spiny neurons (cyan), glutamatergic pyramidal neurons (red) and GABAergic cortical interneurons (blue). A minor fraction of single nucleotide polymorphisms associated with increased risk for SCZ may affect oligodendrocyte precursor cells (OPCs) and oligodendrocytes and their function (yellow). Nonetheless, oligodendroglia intensively interact with pyramidal projection neurons and cortical interneurons at the level of myelination and metabolic support. Myelination of long-range projection neurons supports the connectivity between brain regions (‘macro-connectivity’). Myelination and trophic support of interneurons may support the function of local circuits (’micro-connectivity’). Therefore, we hypothesize that the disturbed functional connectivity in SCZ results from the interaction of cell types and mechanisms where the primary effects occur (e.g. directed at synapses in glutamatergic and GABAergic neurons), with secondary effects on OPCs and oligodendrocytes finally causing white matter alterations. It is tempting to speculate that (i) positive symptoms are likely more connected to the dysfunction of dopamine-responsive medium spiny neurons and (ii) excitation-inhibition dysbalances of cortical glutamatergic and GABAergic neurons and disturbed connectivity, including oligodendroglia functions, may rather be associated with higher order cognitive impairments and negative symptoms

The contribution of brain cell types to SCZ was recently addressed in a sophisticated analysis combining the most comprehensive list of SCZ GWAS hits with a series of single cell (sc) and single nucleus (sn) RNAseq analyses from mouse and human brain tissues, which provide dramatically enhanced cell-type resolution.^46^ In contrast to the previous, above mentioned pathway analyses, which relied on lists of expert curated cell type markers,^74,75^ Skene et al.^46^ computed cell type-specific gene expression metrics on the basis of scRNAseq and snRNAseq data. Moreover, they weighted risk gene contributions for genetic association probabilities on the basis of GWAS data. With this approach, the group made several observations with respect to brain cell types associated with SCZ: (i) cell-type profiles from mature (e.g. pyramidal and cortical interneurons) but not from immature cell types (e.g. neuronal progenitors, radial glia) were associated with SCZ; (ii) a dedicated set of mature neuronal cell types (medium spiny neurons, cortical and hippocampal glutamatergic projection neurons and cortical GABAergic interneurons) showed greater association with SCZ than any other neuronal and glial cell type; and (iii) human, but not mouse, reference mRNA cell type profiles indicated that OPCs and OLs also showed a moderate enrichment. These observations likely helped to shape the current hypotheses on SCZ aetiology, which highlight late cortical developmental processes, including microcircuit maturation (‘micro-connectivity’), rather than early neurodevelopmental processes. Moreover, the association of gene sets from cortical and hippocampal projection neurons support the idea that the connectivity between brain regions (‘macro-connectivity’) may also be critical. Because both ‘micro-connectivity’ and ‘macro-connectivity’ essentially depend on trophic support and myelination (see above), it appears possible that the ‘intermediate’ enrichment of OPC-/OL-specific gene sets further supports these assumptions. Although the analyses by Skene et al.^46^ made use of the most comprehensive data sets available so far, the conclusions of their study are not definite because of some technical limitations: (i) non-cell type-specific gene sets may operate to a variable, disease-relevant extent in different cell types (e.g. alteration of gene expression by the H3-K4 methylation pathway, see above); (ii) so far, human brain region- and cell type-specific RNA reference profiles are only available as nuclear transcriptomes, which generates a bias on transcripts involved in cellular processes, such as those associated with dendrites and/or synapses (compartmentalized transcription has also been described in cellular processes of OLs^76,77^); and (iii) although scRNAseq and snRNAseq data sets offer an unprecedented cell type definition and resolution, in general the depth of sequencing per individual transcriptome is low and low abundance transcripts coding for transcription factors or signalling components, for example, may be underrepresented. Besides these limitations, the above findings give the impression that common risk variants of SCZ primarily target dysfunctions of striatal and cortical neurons, which may cooperate with a minor fraction of risk factors operating in OPCs/OLs to disturb the local and remote connectivity of brain networks (Fig. 1).

## Phenomics-based patient stratification

On the phenomics side, the use of a case-control condition as a target phenotype has inherent limitations, including patient heterogeneity and overlap of the symptom spectrum. A potentially more promising strategy might be to use alternative or additional (sub)phenotypes within these patients that may be closer to the aetiological roots of psychotic disorders.^78^

### Cognition-based patient stratification

Cognitive impairments are a key aspect of SCZ^79^ and an attractive but still mainly untapped target for drug development.^29^ Several aspects of cognition, including working memory, attention and executive performance, are affected in SCZ and are already detectable in early-onset SCZ and high-risk individuals.^80^ Moreover, cognitive impairments that occur during the first episode are very stable and improvements or deteriorations are hardly detectable in SCZ patients even after 10 years.^81,82^ Besides the clinical aspects of cognitive impairment as one of the main symptoms in SCZ, there is also a genetic relationship between cognition and SCZ risk genes. Thus, an increased PRS for SCZ is associated with reduced spatial visualization (measured by a block design test) in a cohort of patients, relatives and healthy controls.^83^ Assessment of cognitive abilities with the Brief Assessment of Cognition in Schizophrenia (BACS) also showed an association of PRS with cognitive performance in both SCZ patients and healthy controls.^84^ Besides a general association between increased polygenic risk for SCZ and impaired cognition, a large study identified 21 independent SCZ risk loci that influence cognitive functioning.^85^ Furthermore, cognitive performance was found to be slightly worse in adult healthy carriers of risk copy number variations that are associated with SCZ than in healthy non-carriers.^86,87^ On the functional level, a higher PRS is associated with hypoactivity in the prefrontal cortex during working memory tests in healthy controls.^88,89^ Most recently, the largest GWAS on human intelligence performed so far identified hundreds of genes involved in this trait.^90^ The same study reported a negative genetic correlation between intelligence and SCZ (*r*_g_ = −0.21, *P* = 3.82 × 10^−17^). Remarkably, most prominent gene sets associated with cognition are enriched in medium spiny neurons and hippocampal projection neurons.^90^

In summary, this body of clinical and genetic evidence suggests that cognitive dysfunction in SCZ is an integral part of the disorder rather than a consequence of it. Therefore, using cognition as a stratification criterion for patient selection has the potential to capture key elements in the genetic/molecular architecture of SCZ that are measurable in cellular systems. The results of the aforementioned studies based on SCZ PRS and cognition further support this notion. However, the amount of variation in cognition explained by the SCZ PRS is very modest, which strongly advises against using it as the only patient selection criterion. Although the SCZ PRS is currently the best measure of genetic risk available, the score alone may not yet allow a proper stratification of patient subgroups for decisions regarding iPSC generation and follow-up analyses.

### Imaging-based patient stratification

Patients with early-onset and first-episode SCZ already display widely distributed reduced WM homogeneity, as indicated by a substantial reduction of fractional anisotropy in most brain areas, including several fibre tracts; this reduced WM homogeneity has been associated with disturbed higher order cognitive function.^53,91–93^ These findings were recently verified by the largest imaging study conducted so far, which analysed 29 independent and international imaging studies that included a total of 4322 individuals with SCZ.^94^ Moreover, individuals at high risk for psychosis already display altered WM integrity similar to the pattern of first-episode patients.^95^ Systematic reviews and meta-analyses substantiate a pattern of WM impairments and structural dysconnectivity in patients with early-onset SCZ, drug-naive patients and clinical high-risk individuals^96^ and link disturbed WM in first-episode SCZ to cognitive deficits.^97^ A pioneering study identified a strong association between two risk SNPs in genes important for OL function (*MAG* and *OLIG2*), the integrity of WM tracts and cognitive performance.^98^ The use of genome-wide analyses will likely provide stronger confidence about which genetic factors and pathways may link WM deficits with cognitive impairments in SCZ. In terms of identifying novel drug targets, understanding the biological pathways that modulate the course of the disease over time will provide additional opportunities beyond current genetic approaches.^99^

**Fig. 2:**
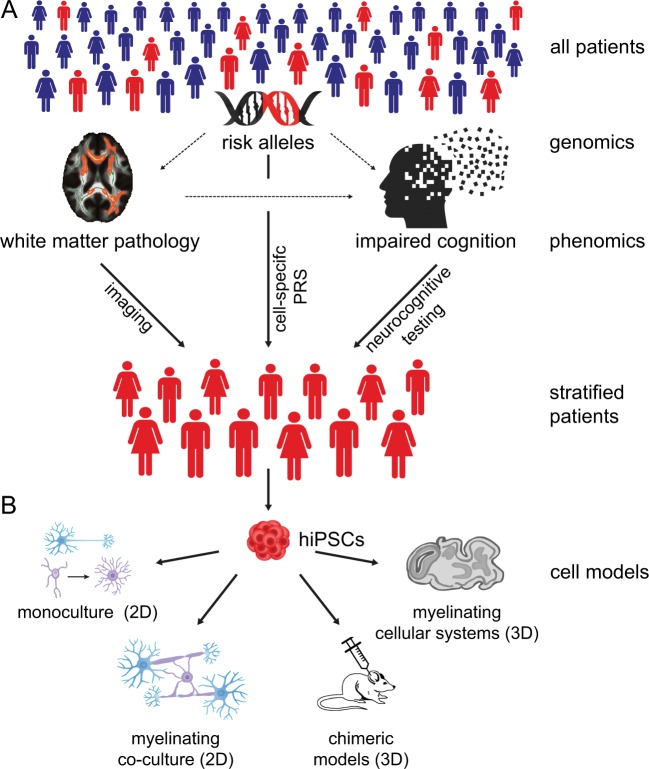
Principals of patient stratification for subsequent human-induced pluripotent stem cell (hiPSC-)-based cellular disease modelling combining genetics, white matter pathology and cognitive impairments. **a** Subsets of SCZ risk genes (as indicated by the red part of the DNA symbol) impair cognitive performance. Red human icons illustrate such risk gene carriers. Recent evidence suggests that the effect of these ‘cognitive’ risk genes is at least in part connected to white matter pathology. Sufficient patient stratification is needed to reveal the underlining mechanisms of white matter pathology. Clinical deep phenotyping, with a focus on neurocognitive testing, combined with imaging of white matter is probably a suitable approach to identify the corresponding subgroup of patients. Additional stratification based on cell-specific PRSs might further increase stratification precision. **b** hiPSC technology enables the generation of a toolbox of patient-derived cell systems. Monocultures of glial cells and neurons and myelinating co-culture systems may simulate disease-relevant aspects of SCZ in 2D and 3D cellular systems in vitro. Moreover, hiPSC-derived cells can be tested in chimeric mouse models in vivo. NB: The illustrations of the ‘chimeric mouse’ and the DNA ‘risk alleles’ have been published previously^100^

Accumulating evidence suggests that WM pathology and functional dysconnectivity are key aspects of SCZ pathophysiology that contribute to cognitive impairments. Thus, the combined use of phenotypes related to cognition and neuroimaging holds great potential for patient stratification and may be less affected by experimental noise than current genetic classifiers. A critical issue for improving selection strategies even further in the future could be to include environmental stress factors, if available. The additional stratification tools, i.e. cognitive and imaging-related phenotypes and environmental stress factors, might help to identify representatives of (sub)groups with verified WM pathophysiology and subsequently to investigate their underlying mechanisms and their impact on cognition in SCZ, e.g. with hiPSC-based cellular modelling (Fig. 2a).

## Generation of hiPSC-based neurobiological cell systems

Until recently, most insights into psychiatric diseases, including SCZ, have been generated from postmortem tissue samples, brain imaging, genetic and pharmacological studies and animal models. Cellular reprogramming methods now provide a new opportunity to model the complex polygenetic conditions in diseases such as SCZ by generating patient-derived hiPSC-based systems.

### Generation of hiPSCs from patients

The first successful reprogramming into iPSCs was performed with murine fibroblasts^101^ and was awarded the Nobel Prize in Physiology or Medicine 2012. Multiple subsequent studies in humans successfully reprogrammed diverse somatic cell types that are more accessible than fibroblasts, such as keratinocytes,^102^ urine cells^103^ and (cord) blood samples.^104–108^ Peripheral blood mononuclear cells (PMBCs) can be reprogrammed even after cryopreservation.^109^ Initial reprogramming methods relied on constitutive retroviral and lentiviral expression systems that were limited by insufficient genomic integration and insertional mutagenesis.^110^ In recent years, integration-free reprogramming methods have been developed on the basis of adenovirus,^111^ mRNA,^112^ sendai virus^113^ and episomal vectors.^114^ Episomal reprogramming is highly reliable for different somatic cell lines, and the rapid loss of the reprogramming factors from the host genome over short time periods makes this method highly attractive.^115^ For translational clinical research, it is crucial to choose the best reprogramming method and the most suitable primary cell type, i.e. the cell type that is most easily accessible and can be stored for long periods.^116^ Because PMBC sampling and cryopreservation are standard procedures and less invasive than skin biopsies, PBMCs represent the best choice for human cell-based studies on psychiatric diseases. Moreover, setting up large PBMC repositories for clinical studies is a routine procedure that might allow post hoc access to patient data, e.g. for validation purposes beyond the ‘subgroup representatives’ selected in first-round reprogramming. Thus, the use of PBMCs (e.g. CD34+ hematopoietic stem cells and erythroid progenitor cells)^117–119^ and episomal reprogramming enables hiPSCs to be generated in a safe, efficient and scalable approach.

Once generated, hiPSCs can be differentiated in principle to all specific brain cell types by providing a lineage-specific chemical environment and/or transient overexpression of transcription factors that determine the cell lineage. Protocols have been developed for hiPSC-derived neuronal stem cells (iPSC NSCs), hiPSC-derived glutamatergic and GABAergic neurons (hiPSC neurons), hiPSC astrocytes, hiPSC OPCs and hiPSC OLs (see below). The generation of disease-relevant cell types represents a major step forward in studying aspects of the neuronal and glial contribution to SCZ in 2D or 3D cellular systems without losing the genetic complexity (Fig. 2b).

### Differentiation of hiPSCs to neuronal cells

The most prominent cell types with significant importance in the modelling of psychiatric diseases are the hiPSC neurons. By using different protocols, glutamatergic excitatory neurons can be generated with similar properties as those of the cortex^120^ and hippocampus.^121^ Specific overexpression of certain transcription factors alone,^122,123^ or in combination with small molecules,^124^ enables more homogeneous and scalable populations of glutamatergic iNeurons to be obtained. Besides excitatory neurons, stem cells can also be differentiated into cortical GABAergic interneurons similar to medium spiny striatal interneurons^125^ or Parv+ interneurons.^126,127^ For more details, we refer readers to comprehensive reviews on the differentiation of iPSC-derived neurons^120^ and iPSC-derived astrocytes^128^ or microglia.^129^

### Differentiation of hiPSCs into the oligodendroglial lineage

The initial approaches employed to generate ‘first-generation’ OPCs from stem cells used small molecule cocktails that inhibited or activated restrictive signalling pathways. These approaches are comprehensively reviewed in detail elsewhere.^130–132^ In general, the main disadvantages of such chemical differentiation approaches are the very protracted protocols, which can take up to several months and are expensive, the high variability in the generated OPCs and the limited numbers of differentiated cells produced with the desired properties. Consequently, these approaches are hardly suitable for diagnostic and empirical perturbation purposes. Another strategy to generate OPCs/OLs is the forced overexpression of lineage-determining transcription factors (as pioneered for iNeurons, see above). Rodent fibroblasts have been successfully trans-differentiated in this way.^133,134^ Furthermore, several combinations of certain transcription factors can differentiate hiPSCs or predifferentiated hiPSC NSCs into iOPCs and iOLs relatively quickly and efficiently (Table 1).

**Table 1:** Fast differentiation of human-induced pluripotent stem cells (hiPSCs) or predifferentiated hiPSC neuronal stem cells (NSCs) to induced oligodendrocyte precursor cells (iOPCs) or induced oligodendrocytes (iOLs) by using selected expression of transcription factors

Cells used	Transcription factors	Generated cell stage	Days	References
hiPSCs	SOX10, OLIG2	iOPC (PDGFRα+/O4+)	14	Li et al.^135^
		iOL (CNP+)	42	
hiPSC NSCs	SOX10, OLIG2, NKX6.2^a^	iOPC (O4+)	28	Ehrlich et al.^136^
		iOL (MBP+/CNP+)	35	
hiPSCs	SOX10, OLIG2	iOPC (PDGFRα+/O4+)	10	Pawlowski et al.^137^
		iOL (MBP+/PLP+/CNP+)	20	
hiPSCs	NKX2.2	pre-iOPC (PDGFRα+)	30	Rodrigues et al.^138^
		iOPC (O4+)	55	
hiPSCs	SOX10^a^	iOL (O4+/MBP+/PLP+)	22	García-León et al.^139^

^a^Best combination of transcription factors to reach (different) endpoint

Thus, several combinations of stage-specific transcription factors seem to be sufficient to differentiate hiPSCs into the OPC/OL lineage by directed overexpression. The studies listed in Table 1 used different combinations of transcription factors and applied different cell media and supplement compositions. Although the major focus of Pawlowski et al. and Rodrigues et al. was to generate and characterize the iOPC stage,^137,138^ Li et al., Ehrlich et al. and García-León et al. tried to reach the iOL stage as part of the main protocol.^135,136,139^ Indeed, it is unclear whether protocol differences may have affected the properties of iOPCs or iOLs, e.g. with respect to kinetics and the efficiency of differentiation and myelination. Thus far, no study has compared the protocols by applying a comprehensive battery of molecular and morphological assessments, but such a study could help to identify potential (dis)advantages of the different conditions.

### Myelinating 2D culture systems

hiPSC OPCs and hiPSC OLs not only express state-specific markers, but are also able to myelinate axon-like structures,^136^ human foetal neurons^140^ and hiPSC neurons.^136,139^ They can be used in co-culture systems to investigate axoglial interactions or, for example, to assess pro-myelinating drugs with potential relevance also for SCZ. After pioneer experiments in mouse pluripotent epiblast stem-cell-derived OPCs,^141^ studies have also shown that clobetasol, miconazole and pranlukast promote myelination of hiPSC OPCs^136,139,141^ (Fig. 2B).

### Myelinating 3D culture systems

As a highly advanced in vivo test system, hiPSC OPCs have been used in human-mouse chimeric models. hiPSC OPCs have the capacity to myelinate mouse brain slices ex vivo^139^ and brain regions in living animals.^136,138,140^ Chimeric hiPSC-based systems thus allow glial pathophysiology to be investigated in complex organisms up to the behavioural level (Fig. 2B).^47^ Newly developed 3D cell culture systems enable disease biology to be studied at the circuit level in an experimentally accessible and complex cellular environment.^142,143^ The 3D organoid culture systems, termed cerebral organoids, recapitulate some critical features of human cortical development. To date, maturation of circuits by spine pruning or myelination cannot be studied in cerebral organoids because of the lack of OLs and microglia.^144^

Another, technically ‘less challenging’ approach uses hiPSC-derived cortical spheroids, which consist of a cerebral cortex-like structure and include astrocytes and interneurons.^145^ Recently, Madhavan et al. generated hiPSC-derived ‘oligocortical spheroids’,^146^ in which 20% of the contained cell were part of the oligodendroglial linage after 14 weeks of maturation. Early-stage myelination of neurons occurred after 20 weeks, but myelin maturation, refinement and myelin compaction were not complete until 30 weeks of maturation. Thus, higher level processes such as myelination can be studied in 3D hiPSC-derived cerebral spheroids. However, the time and costs of generating them are still quite challenging.

An additional, less complex but much faster approach is the generation of an hiPSC-derived 3D brain microphysiological system (BMPS).^147^ The key accelerating step in this approach is the pre-differentiation of hiPSC to hiPSC NSCs before the 3D generation of the BMPS. In just 2 weeks Pamies et al.^148^ generated a BMPS that contained GABAergic, dopaminergic and glutamatergic neurons, astroglia and oligodendroglia. Intriguingly, this approach allowed Pamies et al. to observe complex processes such as synaptogenesis and myelination within only 4 weeks, and 42% of axons were myelinated after 8 weeks. The above mentioned pioneering studies have limitations regarding intra- and inter-individual variability, cellular robustness, reproducibility, scalability and affordability. However, the field of 3D cellular systems, as well as the whole-hiPSC field, is rapidly evolving. Thus, in the near future we might expect more robust protocols for 3D hiPSC-based cell systems that contain OPCs and OLs (Fig. 2B).

## Recent observations and challenges of patient-specific neurobiological test systems

The landmark study by Brennand et al. in 2011 that characterized hiPSC neurons from SCZ patients was performed in a mixed population of glutamatergic, dopaminergic and GABAergic neurons that showed decreased neuronal connectivity, decreased neurites and decreased levels of post-synaptic protein PSD95.^148^ Recent studies have focused more on specific neuronal subtypes, such as pyramidal cortical interneurons, cortical interneurons and dentate gyrus (DG) granule neurons; these studies are reviewed in detail elsewhere.^20,149^

### Disturbed OPC/O function and impaired myelination in SCZ-relevant iPSC cell systems

In contrast to the large number of studies on hiPSC-derived neurons, so far only a few studies have investigated the oligodendroglial impact in SCZ-related iPSC models. Chen et al. focused on the known schizophrenia risk gene FEZ1 in murine and human iPSC-derived oligodendroglial cells and showed that FEZ1 knockdown impaired OL development^150^. Additional analysis revealed that potential SCZ-relevant pathways governed FEZ1 expression and post-transcriptional stability^150^. Lee et al. used hiPSC neurons and hiPSC OPCs from two individuals with a large (289 kb) heterozygous deletion in CNTNAP2 that affected exons 14–15, whereby both the patient with SCZ and the patient’s healthy father, who carried the allele, were heterozygous.^151^ The research group showed that the expression of exons 14–15 was significantly decreased in hiPSC-derived neurons and OPCs, but not in fibroblasts, whereas the expression of other exons was upregulated, indicating cell-type-specific mechanism.^151^ Another family-based approach investigated the contribution of two rare missense mutations in CSPG4 (A131T and V901G).^152^ CSPG4 codes for NG2, a marker of proliferating OPCs, which are frequently termed NG2-glia.^153,154^ hiPSC OPCs derived from CSPG4 mutation carriers revealed dysregulated posttranslational processing, different cellular NG2 location, impaired OPC survival and reduced differentiation to mature OLs.^152^ Transfection of the two risk variants into OPCs generated from healthy non-carrier siblings revealed similar deficits, underlining the contribution of the described mutations to the OPC pathology. By using diffusion tensor imaging (DTI), the investigators observed impairments of white matter integrity in affected mutation carriers but not in unaffected siblings or the general population.^152^ Interestingly, no pathological signs were detected in carrier-derived hiPSC neurons, underlining the OPC-specific effect of the CSPG4 mutations.^152^

These studies were either prototypical, hypothesis-driven or single-gene interference approaches or they evaluated ‘rare’ variants that are thought to reflect high-penetrance and ‘high-impact’ mutations; thus, the studies are limited in their dissecting mechanisms and restricted to the dysfunction of a single gene or locus. The generation of iPSCs and derived cell systems from patients who are carriers of polygenic risk assemblies could enable a deeper understanding of common molecular roots of SCZ in a given cell type, such as OLs. Moreover, the differentiation protocols applied in the studies mentioned above were solely based on small molecule differentiation protocols, which may be compromised by having a higher level of variability than directed protocols. Nonetheless, direct cell lineage conversion may possibly override subtle developmental deficits, which may be better accessible with chemical protocols (see below).

In the seminal study by Windrem et al., glial precursor cells, which could mature into both oligodendroglial and astroglial lineage cells, were generated from patients with childhood-onset SCZ and unaffected controls.^47^ SCZ-derived precursor cells displayed impaired glial maturation, altered transcriptomic signatures in vitro and hypomyelination; when grafted into brains of immune-deficient mice, the mice showed psychosis-related behaviours, such as deficits in sensory motor gating.^47^ This study provides the strongest evidence so far that glial precursor cells have a cell-autonomous effect that is thus a potentially ‘primary’ cause of the disease or at least contributes to it. It seems likely that the majority of SCZ-relevant endophenotypes detected by Windrem et al. were caused by glial dysfunction and related to disturbed myelination and impaired functional connectivity. Nonetheless, more studies are needed to dissect the relative impact of the cell-type-dependent risk in individual patients.

### Challenges for the future of disease-relevant cell systems

To date, hiPSC-based studies in psychiatric disorders have mainly been performed on adherent monolayer cultures and have focused on cell-autonomous molecular and cellular abnormalities. Most protocols applied to generate neuronal cell types also contained cells of mixed temporal and spatial identities and other cell types of glial and non-neural origin.^155^ The large heterogeneity of these mixed cell cultures leads to a substantial and hard-to-control level of variability, even when the cells are obtained from the same individual, and decreases the confidence and robustness of the results (i.e. observed SCZ vs. control samples). One very costly and potentially impracticable strategy to tackle this issue is to increase the technical and biological replicates.^156^ Another, possibly more feasible strategy to increase levels of reliability is to reduce the cellular heterogeneity of hiPSC-based cell systems. This may be possible by the directed generation of cell types via overexpression of defined cell- and stage-specific transcription factors, as described above. These paradigms may bypass certain maturation processes, which might be a disadvantage when studying psychiatric diseases, in which neurodevelopmental aspects are hypothesized to play a role, such as SCZ. As regards SCZ, however, recent analyses of GWAS data did not provide evidence for a strong association between early developmental cell stages and the disease (see above^46^).

Although great progress has been made with 3D cell-based neurobiological model systems, they still have major limitations. Current protocols for organoids mimic early neurodevelopment but lack mature OLs and do not develop to postnatal maturation stages, including myelination and synapse pruning. However, recent landmark studies have managed to generate myelinating spheroids and brain microphysiological systems and we can expect more elaborated protocols in the future. One of the current challenges is to develop procedures to establish more mature 3D cell systems. For both 2D and 3D cell systems, scalability is an important issue to enable genetic and chemical screenings at high throughput. Another important topic is intra- and inter-patient variability.^157,158^ To increase reliability, multiple hiPSC clones should be analysed. An alternative strategy could be to initially focus on a pool of clones and to subsequently validate the obtained effects in individual cell clones to substantiate robust findings. In any case, modern array-based genotyping tools should be applied to verify the genomic integrity and genotype beyond karyotyping at the chromosome level.

Even if further progress is made in 2D and 3D cellular systems, they will always be limited regarding construct validity in brain disorders where circuit-level dysfunctions precipitate behavioural and cognitive deficits. We must accept that even patient-specific cellular models, which reflect the genetic background of an individual, only allow us to study certain disease-associated endophenotypes, such as axonal support or multiple aspects of myelination, in isolation. The possibility to generate iOLs and myelinating 3D systems with patient-derived OLs simply will expand the experimental repertoire in psychiatric research.

## Patient selection as a pre-requisite to overcome the bottleneck of limited hiPSC-based cellular test systems

One of the major questions that researchers face is how to select representatives of subgroups from whom hiPSC will be derived for empirical testing. An approach that may yield successful results is to use different analytical methods to select individuals according to phenotypical characteristics. This approach requires the availability of deeply phenotyped samples. Whereas selection based on a simple clinical feature may have limitations, clustering individuals according to different clinical subdomains (e.g. psychopathology, neurocognition or response to treatment) and imaging (e.g. DTI) may yield more valid clusters of individuals with an enhanced biological validity, similar to the Research Domain Criteria (RDoC) concept.^159,160^ Moreover, including longitudinal information into such a clustering will undoubtedly enrich the validity of the clusters in terms of disease progression/trajectory and potentially also treatment response. However, an important limitation of this approach is the high degree of uncertainty regarding the role of the environment in determining the clinical and neurocognitive profile of a patient. Another method of selecting patients is to leverage the current knowledge on the genetic architecture of complex disorders, such as SCZ.^78^ Given their polygenic nature, one could select those individuals who are carriers of the largest/lowest genetic burden on the basis of their individual PRS and subsequently analyse the characteristics of their cellular systems.^78^ Such a polygenic score could also be biologically informed on the basis of pathways or cell-type information (cell-type-specific PRS). This approach would be extremely relevant if the hypotheses and cellular systems are based on a specific cell type (Fig. 2B). Here again, however, caution is advised when using the PRS as a stratification criterion, given the modest amount of variation in clinical diagnosis explained by SCZ PRS.

**Fig. 3:**
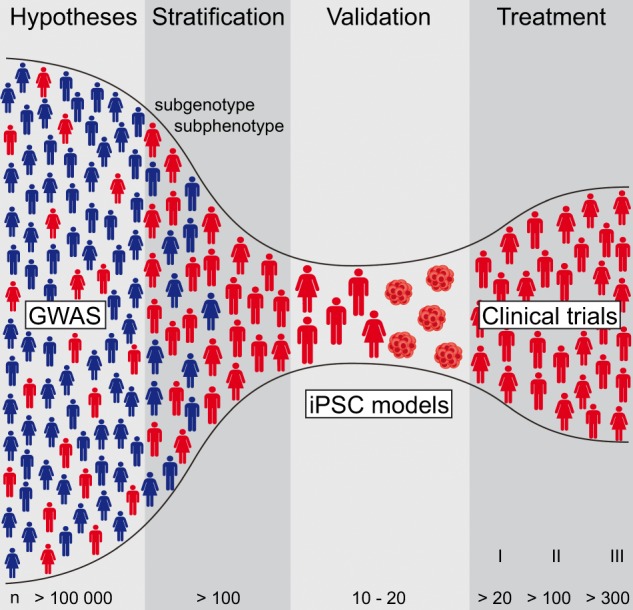
hiPSC models are the bottleneck for molecular validation of hypotheses driven by genome-wide association studies (GWASs). Analyses based on GWASs (*n* > 100,000 individuals) in combination with clinical investigations allow for definition of causal hypotheses in complex diseases, such as SCZ. The next major step is sufficient patient stratification for the genetic subtype and corresponding subphenotype (*n* > 100). Subsequent iPSC reprogramming from the identified representative patients remains the bottleneck because the process is so cost and labour intensive. hiPSC models enable experimental validation and evaluation of GWAS-driven hypotheses. These patient-derived cell systems allow researchers to screen treatment options and pave the way for new therapies that can be introduced after being verified in clinical studies with increasing numbers of patients; these studies are best performed in patient subgroups that align with the initial stratification strategy

Imaging techniques might allow patients with impaired white matter tract integrity to be included; these patients might hold the promise of having oligodendroglial pathology, which could be confirmed by combining imaging with cognitive profiling, for example. The subsequent generation of hiPSC cell systems will be very labour- and cost-intensive and represents the bottleneck in the process. This strongly argues for a stringent, at best hypothesis-driven pre-selection of relevant subgroup representatives, which might enable investigators to reveal corresponding molecular mechanisms in SCZ. There is a chance that hiPSC technology might be a future key technology to find new cellular targets by performing genetic and chemical perturbation screenings with relevance for a given subgroup and/or trait, even with very limited numbers of test systems. Ideally, subsequent validation of candidate drugs in clinical trials and approved treatment should be restricted to those patients who fit the initial classifiers used for ‘empirical validation’, such as white matter abnormalities combined with cognitive deficits (Fig. 3).

## Outlook

The advent of hiPSCs combined with the generation of in principle any neuronal or glial cell type holds great potential for ex vivo disease modelling. A wide range of disease modelling iPSC-based systems have been developed, ranging from 2D monocultures to complex 3D myelinating multicellular systems, and we can expect that many more protocols and conditions will be explored in the future. However, the high costs and cellular variability are important limitations and represent crucial challenges, as does the issue of reproducibility. Besides these technical limitations, meaningful patient stratification is another important issue to be addressed in the future. Despite important limitations and challenges, in principle hiPSC-based cellular disease modelling offers a possibility to address cellular phenotypes in patients with SCZ, whereas retaining the genetic background of each individual. This approach, however, requires stringent experimental proof of the stability of the respective genotype throughout the process of reprogramming, cell-line establishment and differentiation. Unfortunately, experiments to prove stability have not yet been implemented in the field. Epigenetic signatures may also introduce an as yet unknown layer of variability. Nonetheless, the decrease in costs for genomic and epigenetic profiling technologies should allow the field to cope with these sources of variability when setting up batteries of iPSC lines that may complement clinical studies. If successful, personalized strategies that include these new technologies might help to address old questions and reveal new molecular pathways that contribute to neuropsychiatric diseases such as SCZ^161^ and might also enable targeted drug development.^162^ The field of hiPSC technology in neuroscience is rapidly evolving and constantly progressing. We are witnessing a new era of psychiatry research that brings new challenges, new solutions and new possibilities.

## Data Availability

Data sharing are not applicable to this article because no data sets were generated or analysed.
